# Expression of Stanniocalcin-1 and Stanniocalcin-2 in Laryngeal Squamous Cell Carcinoma and Correlations with Clinical and Pathological Parameters

**DOI:** 10.1371/journal.pone.0095466

**Published:** 2014-04-17

**Authors:** Han Zhou, Ying-Ying Li, Wei-Qiang Zhang, Dan Lin, Wei-Ming Zhang, Wei-Da Dong

**Affiliations:** 1 Department of Otorhinolaryngology, The First Affiliated Hospital, Nanjing Medical University, Nanjing, China; 2 Department of Pathology, The First Affiliated Hospital, Nanjing Medical University, Nanjing, China; University of North Carolina School of Medicine, United States of America

## Abstract

**Background:**

Stanniocalcin-1 (STC1) and stanniocalcin-2 (STC2) are secreted glycoprotein hormones involved in various types of human malignancies. The roles of STC1 and STC2 in laryngeal squamous cell carcinoma (LSCC) remain unknown. We investigated correlations between STC1 and STC2 expression and clinicopathological or prognostic factors in LSCC.

**Methods:**

Pre-surgical peripheral blood samples were collected between 2012 and 2013 from 62 patients with LSCC. Quantitative RT-PCR analysis was performed to examine mRNA levels of *STC1* and *STC2*. Immunohistochemistry was performed to retrospectively analyze 90 paraffin-embedded LSCC tissue samples, which were obtained from patients who received surgery between 2006 and 2009. These patients did not have histories of treatment or malignancies. Univariate analysis of patient survival was performed by the Kaplan–Meier method. Multivariate analyses were performed with the Cox proportional hazards model.

**Results:**

The relative mRNA levels of *STC1* and *STC2* in peripheral blood were significantly greater in LSCC patients than those of healthy volunteers (both *P*<0.05). STC2 protein expression in tumor tissues was associated with invasion into the thyroid cartilage, T-Stage, lymphatic metastasis, clinical stage, and pathological differentiation (all *P*<0.05). In addition, STC2 protein expression was an independent prognostic factor for overall survival in patients with LSCC (*P* = 0.025). In contrast, STC1 expression only correlated with clinical stage (*P* = 0.026) and was not an independent or significant prognostic factor.

**Conclusions:**

Circulating *STC1* and *STC2* mRNA are potentially useful blood markers for LSCC. Our results strongly suggest that the STC2 protein, but not STC1, may be a valuable biomarker for LSCC malignancies and a prognostic marker for poor outcome following surgery. Future studies should examine STC2 as a novel molecular target for the treatment of LSCC.

## Introduction

Laryngeal squamous cell carcinoma (LSCC) is one of the most common upper aerodigestive tract epithelial malignancies in the world [1]. LSCC can develop in any part of the larynx, including the glottis, supraglottic, and subglottic areas. In China, the incidence of LSCC has gradually increased over the past several decades. Currently, LSCC is the second most common malignant tumor of the head and neck in China [2]. Although patients with LSCC benefit from advanced diagnostic and therapeutic management, survival remains poor and has not improved during the past 30 years [3]. Current tumor-node-metastasis (TNM) staging criteria and differentiation grade are the main factors used to predict outcome in patients with LSCC [4], [5]. However, these parameters do not accurately predict the future course of early-stage LSCC. Moreover, the molecular mechanisms by which LSCC initiates and progresses remain unclear. Therefore, the identification of sensitive and specific molecular markers of LSCC would facilitate early prevention, diagnosis, and treatment. Identification of relevant biomarkers is essential for understanding the pathogenesis of LSCC and for developing new targeted treatment strategies for this tumor type.

Stanniocalcin (STC) was discovered in the corpuscles of Stannius in bony fish, in which help regulate calcium homeostasis [6], [7]. Stanniocalcin-1 (STC1) and stanniocalcin-2 (STC2) are mammalian peptide hormones and are synthesized in almost all tissues. STC1 and STC2 function primarily as paracrine/autocrine factors that regulate various biological functions [8]. Recent studies demonstrate that mammalian STCs (STC1 and STC2) play important roles in tumor progression [8]–[10]. Some clinicopathological studies correlate high expression levels of STC1 and STC2 in human tumor samples with poor prognostic outcomes [8], [11]–[14]. However, the expression levels of STC1 and STC2 in LSCC and correlations with clinical and pathological parameters remain to be determined.

In order to investigate the expression profiles of STC1 and STC2 in LSCC, we assessed their expression levels in formalin-fixed, paraffin-embedded tissue samples obtained from 152 patients. *STC1* and *STC2* mRNA expression levels were also assessed in blood specimens from 62 patients with LSCC and 30 healthy volunteers. Furthermore, possible correlations with clinicopathological and prognostic parameters were analyzed.

## Materials and Methods

### Ethics Statement

This study was conducted in accordance with the Declaration of Helsinki. All patients provided written informed consent for the collection of samples and subsequent analysis. The study was approved by the Institutional Review Board of the First Affiliated Hospital of Nanjing Medical University (2012-NT-027).

### Clinical Samples and Patient Population

Sixty-two patients who were diagnosed with LSCC and treated between January 2012 and April 2013 at the First Affiliated Hospital, Nanjing Medical University, were enrolled in this study. The median age of the patients was 60 years (range, 36 to 87 years), and the study included 58 males and 4 females. Thirty volunteers who visited the hospital for health examinations and appeared to have normal laryngeal mucosae according to laryngoscopic examinations were also enrolled. The healthy controls consisted of 27 males and 3 females with a median age of 59 years (range, 35 to 86 years). Five milliliters of peripheral blood (PB) were collected from each patient before surgery. Mononuclear cells were isolated from PB with lymphocyte-separation media (Sigma, St. Louis, USA). Total RNA was isolated with the RNeasy kit according to the manufacturer’s protocol (Qiagen, Hilden, Germany) and stored at −80°C until further processing.

We also examined data for 90 eligible patients with LSCC (84 males, 6 females) who had detailed clinical records and follow-up data. These patients received surgery in our department from 2006 to 2009 and were followed for at least 2 years or died within two years of surgery. Clinical follow-up data were obtained by telephone or from outpatient records.

All of the patients with LSCC in our study had the following inclusion criteria: no history of radiotherapy or chemotherapy and a diagnosis of primary squamous cell carcinoma of the larynx without other malignancies. Clinical and pathological data were collected for the 152 patients with LSCC (62 patients from 2012–2013 and 90 patients from 2006–2009). Data included age, anatomical site, thyroid-cartilage invasion, differentiation grade, lymph node metastases, treatment, and recurrence. Tumor stage (T-stage) was classified according to the 2002 TNM staging system of the Union for International Cancer Control (UICC). Paraffin-embedded LSCC samples were longitudinally sliced into 4-µm-thick sections for immunohistochemical (IHC) analysis.

### Quantitative RT-PCR Analysis

For real-time quantitative reverse transcriptase-polymerase chain reaction (RT-PCR), reverse transcription was performed with 2 µg of total RNA, an oligo (dT)-18 primer, and M-MLV reverse transcriptase (Takara, Syuzou, Shiga, Japan). The levels of mRNA were determined with an ABI 7500 Real-time PCR System (Applied Biosystems, Foster City, USA) and SYBR Premix EX Taq (Takara). The sequences of the primers were: *STC1*, forward: 5′-TGAGGCGGAGCAGAATGACT-3′, reverse: 5′-CAGGTGGAGTTTTCCAGGCAT-3′; *STC2*, forward: 5′-GGTGGACAGAACCAAGCTCTC-3′, reverse: 5′- CGTTTGGGTGGCTCTTGCTA-3′; and *GAPDH*, forward: 5′-TGAAGGTCGGAGTCAACGG-3′, reverse: 5′-CTGGAAGATGGTGATGGGATT-3′. The PCR mixtures contained 2× SYBR Green Master Mix (Takara), 10 µM primers, and 50 ng cDNA in a 20-µl volume. Reactions were heated to 95°C for 30 seconds followed by 40 cycles of 95°C for 3 seconds and 60°C for 30 seconds. All PCR reactions were performed in triplicate. PCR product specificity was evaluated by melting-curve analysis and by separation on agarose gels. The linearity of PCR amplification was controlled by using five dilutions of cDNA. *STC1* or *STC2* mRNA levels were expressed as fold-increased or fold-decreased relative to *GAPDH* mRNA expression. The mean values of the replicates for each sample were calculated and expressed as cycle threshold (Ct). Gene expression levels were expressed as 2^−ΔCt^, in which ΔCt was the difference between the Ct value of *STC1* or *STC2* and the Ct value of *GAPDH*.

### Immunohistochemical Analysis

IHC staining for STC1 and STC2 was performed with Ultrasensitive immunohistochemistry S-P kits (Maixin Biology Corporation, Fuzhou, China) according to the manufacturer’s instructions. Sections (4-µm thick) were deparaffinized, washed in Tris-buffered saline (TBS), and incubated with 3% H_2_O_2_ in methanol for 20 minutes to block endogenous peroxidases. Sections were then washed with TBS and incubated with 5% skim milk in TBS for 20 minutes. Blocked sections were incubated with antibodies against STC1 (rabbit polyclonal anti-STC1 antibody; Santa Cruz Biotechnology, Santa Cruz, CA, USA) or STC2 (rabbit polyclonal anti-STC2 antibody; Novus Biologicals, Littleton, CO, USA) for 90 minutes at 37°C. A secondary antibody was applied to each section for 10 minutes at room temperature, and the slides were rinsed three times in phosphate-buffered saline (pH 7.4) after each incubation step. The slides were counterstained with hematoxylin, mounted, and observed by light microscopy.

### Evaluation and Scoring of Immunohistochemistry

All IHC slides were independently and blindly assessed and scored by two pathologists with agreement. The final score was an average of the two scores. The staining score for STC1 or STC2 was assessed semiquantitatively according to the percentage of positive staining and the staining intensity. An unequivocal positive reaction was defined as a brown signal in the cytoplasm or on the cell membrane. The staining intensity was scored as 0 (no staining), 1 (weak staining exhibited as light yellow), 2 (moderate staining exhibited as yellow-brown), 3 (strong staining exhibited as brown). The percentage of positive staining was scored as 1, ≤10%; 2, 11–50%; 3, 51–80%; and 4, ≥81%. The final staining score (1 to 7) was calculated as the sum of the staining intensity and percentage score. For statistical evaluation, the expression of STC1 or STC2 was classified as negative (≤10% of the area stained positive regardless of intensity), low (staining score was 3–4 and >10% of area stained positive), or high (staining score ≥5 and >10% of area stained positive) [15].

### Statistical Analysis

Data were analyzed with SPSS19.0 (SPSS Inc., Chicago, IL, USA) and GraphPad Prism version 5.0 (GraphPad Inc., San Diego, CA, USA). Differences in STC1 or STC2 mRNA expression levels in the PB samples of patients with LSCC and that of healthy volunteers were assessed by the Mann-Whitney test. The associations of STC1 or STC2 expression in PB samples or tumors with clinicopathological variables were also evaluated by the Mann-Whitney test. The correlation between STC1 or STC2 expression in tumors and the circulating STC1 or STC2 mRNA level was analyzed by the Spearman’s rank correlation coefficient. Survival times were evaluated by the Kaplan–Meier method with the log-rank test. Multivariate analyses were performed with the Cox proportional hazards model. *P*-value less than or equal to 5 percent was considered to be statistically significant.

## Results

### *STC1* and *STC2* mRNA Expression Profiles in PB from LSCC Patients and Healthy Volunteers

**Figure 1:**
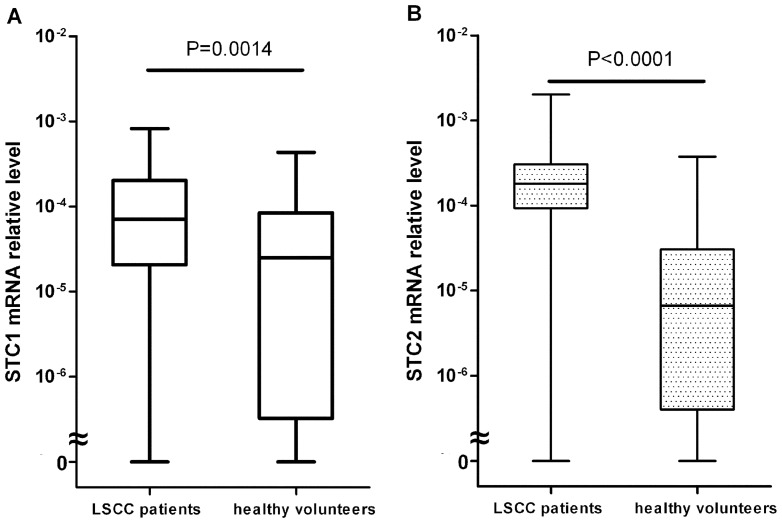
STC1 and STC2 mRNA in peripheral blood from LSCC patients (n = 62) and healthy volunteers (n = 30). Circulating STC1 (A) and STC2 (B) mRNA levels were significantly higher in LSCC patients than those of healthy volunteers as determined by quantitative real-time PCR (both *P*<0.05, Mann-Whitney test). The heavy, black, horizontal line in each box represents the median value.

*STC1* mRNA expression was detected in most of the samples (58 of 62 samples from untreated LSCC patients and 23 of 30 samples from healthy volunteers). As shown in **Figure 1A**, the median value of *STC1* mRNA was 7.08×10^−5^ (range: 0 to 8.22×10^−4^) in blood specimens from LSCC patients and 2.51×10^−5^ (range: 0 to 4.33×10^−4^) in those of healthy volunteers. Similarly, *STC2* mRNA expression was also detected in most of the samples (58 of 62 samples from untreated LSCC patients and 22 of 30 samples from healthy volunteers). The median value of *STC2* mRNA was 1.82×10^−4^ (range: 0 to 2.02×10^−3^) in blood specimens of LSCC patients and 6.65×10^−6^ (range: 0 to 3.76×10^−4^) in those of healthy volunteers (**Figure 1B**). The levels of *STC1* and *STC2* mRNA expression were significantly higher in the PB samples from LSCC patients than those of healthy volunteers (both *P*<0.05).

### Correlation between Circulating *STC1* or *STC2* mRNA Level and Clinicopathological Findings in LSCC Patients

The data on the correlation between circulating *STC1* or *STC2* mRNA level and clinicopathological findings in LSCC patients is listed in **Table 1**. Peripheral *STC1* expression significantly correlated with clinical stage. A possible relationship with thyroid-cartilage invasion was also found (*P* = 0.072). However, circulating *STC1* mRNA levels were not significantly associated with other clinicopathological characteristics. *STC2* expression was significantly correlated with thyroid-cartilage invasion, lymphatic metastasis, and clinical stage. Furthermore, a possible relationship between *STC2* expression and T-Stage was found (*P* = 0.061). There was no significant correlation between *STC2* expression and age, smoking index, drinking, type of laryngeal carcinoma, or pathological differentiation (**Table 1**).

**Table 1:** Circulating *STC1* and *STC2* mRNA levels and clinical characteristics (n = 62).

Variable	Cases	Circulating *STC1* mRNAlevels ×10^−5^	*P*-value	Circulating *STC2* mRNAlevels ×10^−5^	*P*-value
Age (years)			0.636		0.077
<65	43	8.940 (0–82.23)		19.79 (0–201.8)	
>65	19	5.260 (0.870–56.24)		9.740 (0–128.1)	
Smoking index			0.948		0.304
<600	23	5.700 (0–82.23)		14.02 (0–201.8)	
≥600	39	7.350 (0–56.24)		21.15 (0–188.1)	
Drinking			0.721		0.311
None or occasionally	23	9.070 (0–82.23)		14.97 (0–128.1)	
Frequently	39	6.810 (0–59.20)		20.98 (0–201.8)	
Type			0.610		0.130
Glottic	47	6.810 (0–82.23)		16.10 (0–201.8)	
Supraglottic	15	10.99 (1.330–42.54)		31.05 (4.070–188.1)	
Thyroid-cartilage invasion			0.072		0.024
No	47	5.480 (0–56.24)		14.98 (0–188.1)	
Yes	15	14.73 (0–82.23)		40.54 (0–201.8)	
T-Stage			0.113		0.061
T1 and T2	41	5.700 (0–56.24)		14.98 (0–188.1)	
T3 and T4	21	11.87 (0–82.23)		31.05 (0–201.8)	
Lymphatic metastasis			0.250		0.002
No	51	6.810 (0–39.96)		8.780 (0–67.54)	
Yes	11	21.70 (0–82.23)		104.4 (0–201.8)	
Clinical stage			0.031		0.003
I and II	38	5.480 (0–39.96)		14.87 (0–41.06)	
III and IV	24	14.73 (0–82.23)		40.54 (0–201.8)	
Pathological differentiation			0.421		0.626
Moderately and highlydifferentiated	52	6.205 (3.630–82.23)		16.38 (0–201.8)	
Poorly differentiated	10	11.92 (0–56.24)		21.64 (0–128.1)	

Circulating *STC1* or *STC2* mRNA levels are expressed as a median and range.

### Expression of STC1 and STC2 in LSCC Tissues

**Figure 2:**
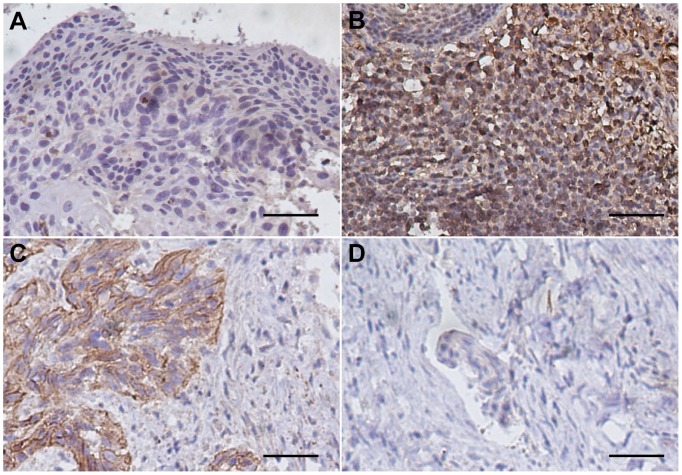
Representative immunohistochemical staining of STC1 in LSCC. Positive immunostaining was observed in the cytoplasm and membranes of tumor cells. (A) Negative expression of STC1 in adjacent non-cancerous epithelial tissues. (B) High expression of STC1 in well-differentiated glottic LSCC. (C) Low expression of STC1 in poorly differentiated supraglottic LSCC. (D) Staining with negative control (phosphate-buffered saline) in LSCC specimens. (magnification 200×, scale bar = 50 µm).

**Figure 3:**
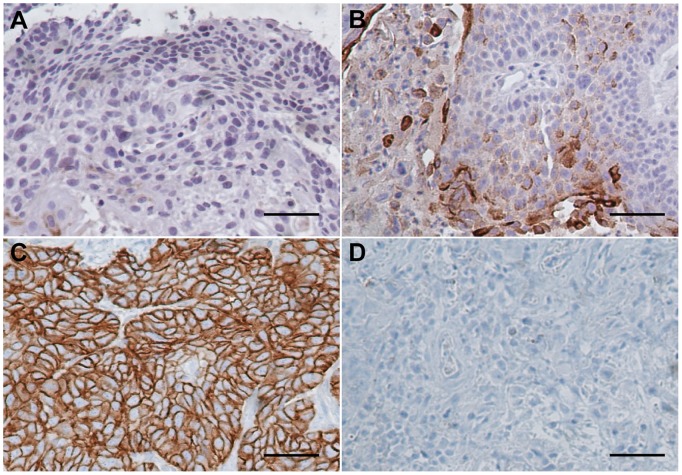
Representative immunohistochemical staining of STC2 in LSCC. Positive immunostaining was observed in the cytoplasm and membranes of tumor cells. (A) Negative expression of STC2 in adjacent non-cancerous epithelial tissues. (B) Low expression of STC2 in well-differentiated glottic LSCC. (C) High expression of STC2 in poorly differentiated supraglottic LSCC. (D) Staining with negative control (phosphate-buffered saline) in LSCC specimens. (magnification 200×, scale bar = 50 µm).

IHC analysis showed that positive staining was observed in most of the tumor tissues and in a small portion of the adjacent normal tissues. Moreover, STC1 and STC2 staining was stronger in LSCC tissues than that of the corresponding normal laryngeal epithelial tissues. STC1 and STC2 were expressed predominantly in the cytoplasm or on the membranes of tumor cells (**Figure 2** **&** **Figure 3** **)**.

### Correlation of STC1 and STC2 Expression with Clinicopathological Findings in LSCC Patients

**Figure 4:**
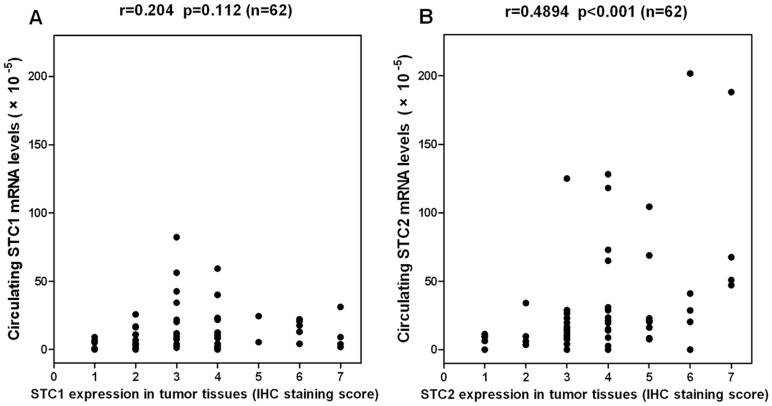
Correlations of circulating STC1 (A) or STC2 (B) mRNA with the corresponding protein in tumors. STC1 or STC2 protein expression was based on the immunohistochemical staining scores (1 to 7) of the LSCC samples. r, Spearman’s rank correlation coefficient.

The data on the correlation of STC1 and STC2 expression with clinicopathological findings in LSCC patients is listed in **Table 2**. STC1 expression in tumor tissues significantly correlated with clinical stage. Potential relationships between STC1 expression and thyroid-cartilage invasion or T-Stage (*P* = 0.086, *P* = 0.083, respectively) were also identified. There was no significant correlation between STC1 expression and age, type of laryngeal carcinoma, lymphatic metastasis, or pathological differentiation. STC2 expression in tumor tissues significantly correlated with thyroid-cartilage invasion, T-Stage, lymphatic metastasis, clinical stage, and pathological differentiation. No significant correlation was found between STC2 expression and age or type of laryngeal carcinoma (**Table 2**). In addition, the expression of STC2 in tumor tissues positively correlated with circulating *STC2* mRNA levels in LSCC patients. However, there was no significant correlation between the levels of STC1 in tumor tissues and circulating *STC1* mRNA levels (**Figure 4**).

**Table 2:** Correlation of STC1 or STC2 expression with clinicopathological variables in LSCC Patients (n = 152).

		STC1, no. (%)				STC2, no. (%)			
Variable	Cases	Negative	Low	High	*P*-value	Negative	Low	High	*P*-value
Age (years)					0.688				0.934
<65	93	31 (33.3)	47 (50.5)	15 (16.1)		19 (20.4)	48 (51.6)	26 (28.0)	
>65	59	16 (27.1)	35 (59.3)	8 (13.6)		12 (20.3)	30 (50.8)	17 (28.9)	
Type					0.620				0.241
Glottic	109	35 (32.1)	58 (53.2)	16 (14.7)		25 (22.9)	55 (50.5)	29 (26.6)	
Supraglottic	43	12 (27.9)	24 (55.8)	7 (16.3)		6 (14.0)	23 (53.5)	14 (32.5)	
Thyroid-cartilage invasion					0.086				0.013
No	115	40 (34.8)	59 (51.3)	16 (13.9)		27 (23.5)	61 (53.0)	27 (23.5)	
Yes	37	7 (18.9)	23 (62.2)	7 (18.9)		4 (10.8)	17 (46.0)	16 (43.2)	
T-Stage					0.083				0.005
T1 and T2	88	31 (35.2)	47 (53.4)	10 (11.4)		23 (26.1)	47 (53.4)	18 (20.5)	
T3 and T4	64	16 (25.0)	35 (54.7)	13 (20.3)		8 (12.5)	31 (48.4)	25 (39.1)	
Lymphatic metastasis					0.585				0.006
No	116	38 (32.8)	60 (51.7)	18 (15.5)		29 (25.0)	59 (50.9)	28 (24.1)	
Yes	36	9 (25.0)	22 (61.1)	5 (13.9)		2 (5.6)	19 (52.8)	15 (41.7)	
Clinical stage					0.026				<0.001
I and II	81	30 (37.0)	43 (53.1)	8 (9.9)		23 (28.4)	43 (53.1)	15 (18.5)	
III and IV	71	17 (23.9)	39 (54.9)	15 (21.1)		8 (11.3)	35 (49.3)	28 (39.4)	
Pathological differentiation					0.698				0.043
Moderately and highly Differentiated	123	38 (30.9)	65 (52.8)	20 (16.3)		27 (22.0)	66 (53.6)	30 (24.4)	
Poorly differentiated	29	9 (31.0)	17 (58.6)	3 (10.3)		4 (13.8)	12 (41.4)	13 (44.8)	

### Correlation of STC1 or STC2 Expression with LSCC Prognosis

**Figure 5:**
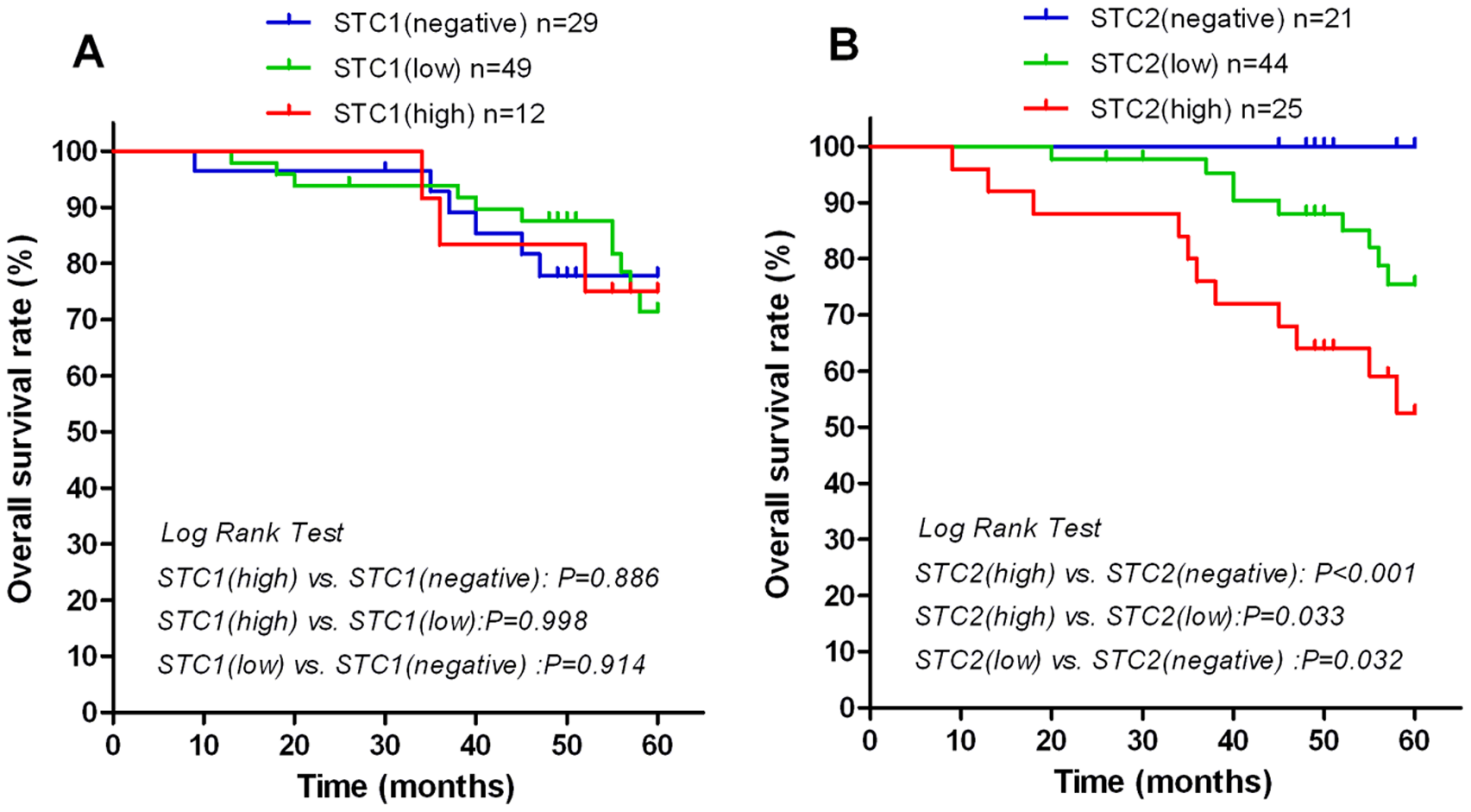
Survival rates according to STC1 or STC2 expression in LSCC patients. The overall survival rate according to STC1 (A) or STC2 (B) expression was plotted by the Kaplan-Meier method. Differences among the three groups (negative, low, or high expression of STC1 or STC2) were evaluated by the log-rank test. Patients with high STC2 expression in tumor tissues had significantly poorer overall survival rates than that of patients with negative or low STC2 expression in tumors.

There were no significant differences in overall survival (OS) rates among LSCC patients with negative, low, or high STC1 expression levels in tumor tissues. In contrast, patients with high levels of STC2 expression in tumor tissues had significantly poorer OS rates than those with negative or low STC2 expression. Similarly, OS rates were lower in LSCC patients with low STC2 than those of patients with negative STC2 expression (**Figure 5**).

Univariate Cox regression analyses determined that thyroid-cartilage invasion, T-Stage, lymphatic metastasis, clinical stage, pathological differentiation, and STC2 expression in tumors were significantly associated with poor OS (all *P*<0.05). These six parameters were included in multivariate analyses. Multivariate Cox analyses demonstrated that lymphatic metastasis, pathological differentiation, and STC2 expression were independent factors with prognostic value for OS (*P* = 0.033, *P* = 0.018, and *P* = 0.025, respectively) in patients with LSCC (**Table 3**).

**Table 3:** Results of univariate and multivariate survival analyses for overall survival by the Cox proportional hazard model (n = 90).

Variable	Categories	RR (95%CI)	*P-*value
**Univariate survival analysis**			
Age (years)	<65/>65	0.392 (0.143–1.080)	0.070
Type	Glottic/Supraglottic	1.939 (0.803–4.681)	0.141
Thyroid-cartilage invasion	No/Yes	4.942 (2.030–12.032)	<0.001
T-Stage	T1 and T2/T3 and T4	3.584 (1.301–9.873)	0.014
Lymphatic metastasis	No/Yes	6.176 (2.453–15.545)	<0.001
Clinical stage	I and II/III and IV	5.925 (1.735–20.231)	0.005
Pathological differentiation	Moderately and highly/Poorly differentiated	5.545 (2.269–13.548)	<0.001
STC1 expression	Negative/Low/High	1.048 (0.536–2.050)	0.890
STC2 expression	Negative/Low/High	3.559 (1.694–7.479)	0.001
**Multivariate survival analysis**			
Lymphatic metastasis	No/Yes	3.064 (1.096–8.566)	0.033
Pathological differentiation	Moderately and highly/Poorly differentiated	3.176 (1.219–8.269)	0.018
STC2 expression	Negative/Low/High	2.477 (1.119–5.485)	0.025

## Discussion

Aberrant expression of STC1 or STC2 correlates with poor prognosis in multiple types of cancer [8]–[14]. We found significantly greater levels of *STC1* and *STC2* mRNA in the PB samples of LSCC patients than those of healthy volunteers. These data are consistent with observations reported for gastric cancer [16], [17], esophageal squamous cell carcinoma [18], and non-small cell lung cancer [12]. Furthermore, we investigated whether STC1 or STC2 levels in LSCC tumors were potential molecular markers of prognosis. We found that overexpression of the STC2 protein in surgically-resected LSCC tissues was associated with features of tumor progression and was an independent prognostic factor for OS. In contrast, STC1 protein expression correlated with fewer features of tumor progression and was not an independent or significant prognostic factor for OS in LSCC.

STC1 and STC2 are involved in tumor progression and metastasis [8]. However, the functional relationships between STCs and human cancers have not been elucidated. Animal and cell culture studies have provided some mechanistic data, which reveal possible functions for STCs in cell growth and apoptosis. The growth-related functions of STCs may be associated with the pro- and anti-apoptotic effects of these proteins. Anti-apoptotic effects have been reported for STC1 in some types of cancer [19], [20], whereas pro-apoptotic effects have been observed in other tumor types [21], [22]. These inconsistencies suggest that STC1 may not be the key regulator of apoptosis in these systems [8]. Instead, the anti- or pro-apoptotic roles of STC1 may vary depending on the source, stage, or subset of cancer cells. Thus, the functions of STC1 in carcinogenesis are diverse and complex. In contrast to STC1, STC2 has been consistently found to have an anti-apoptotic role in cancer cells [23], [24]. The mechanism by which STC2 blocks apoptosis may be related to inhibition of plasma membrane store-operated Ca^2+^entry (SOCE), which protects cells from apoptosis [25]. The progression of solid tumors is usually associated with hypoxia. STC2 is a target gene of hypoxia-inducible factor-1, which stimulates the proliferation of cancer cells during hypoxic conditions [26]. Some mechanistic and functional studies have showed that STC2 expression is induced by hypoxia and contributes to the suppression of apoptosis and survival of cancer cells [23], [26], [27]. These findings suggest that STC2 is a positive regulator of tumor progression.

In some malignant neoplasms, including esophageal squamous cell cancers [13], gastric cancers [11], colorectal cancers [28], and renal cell carcinomas [29], high expression of STC2 correlates with tumor progression and poor prognosis, which is consistent with our findings in LSCC. Current evidence supports the notion that STC2 expression is associated with suppression of apoptosis, and cancer development, and dormancy with later relapse [8], [26], [27], [30]. Similar mechanisms may explain why the cytoplasmic overexpression of STC2 in LSCC cells leads to a more clinically aggressive disease course. These findings suggest that assessing STC2 expression in combination with established clinicopathological features may be valuable for making informed prognostic or therapeutic decisions in patients with LSCC.

A considerable number of studies demonstrate that STC1 promotes tumor migration and invasion [9], [18], [20], [31], with the exception of two studies in breast and ovarian cancer [32], [33]. These inconsistent results may be due to the dynamic and complicated regulatory functions of STC1 in cell growth and apoptosis [8]. Furthermore, the expression of STC1 varies among different tissues and may also vary throughout a given tissue section [7]. Therefore, the functions of STC1 are likely to vary among human tumors. Due to some limitations of this study, the relationship between STC1 expression and LSCC progression and malignancy may not be evaluated precisely. However, our results indicate that the role of STC2 in the development of LSCC is more important than that of STC1.

Tumor cells can disseminate from the primary tumor through the blood or lymphatic circulation during early stages of disease. These cells can survive without causing clinical symptoms and eventually promote recurrence of disease [34]–[36]. Thus, it is imperative to monitor circulating tumor cells as an approach to improve diagnosis or prognosis and develop more effective treatments. Consequently, it is important to identify sensitive and specific blood markers of circulating tumor cells. STC1 and STC2 are involved in various biological mechanisms of tumor progression [8], [11]–[14]. Both STC1 and STC2 are considered to be promising biomarkers, because their mRNA levels are elevated in the PB of cancer patients [12], [16]–[18]. However, little is known regarding the clinical significance of STC1 and STC2 expression levels in PB from patients with LSCC.

Our results showed that circulating *STC1* or *STC2* mRNA levels were significantly correlated with one or more features indicative of worse tumor biology. In comparison with *STC1*, circulating *STC2* mRNA was up-regulated more significantly in LSCC patients than that of healthy volunteers. In addition, circulating *STC2* mRNA was significantly associated with more characteristics of tumor migration and invasion. Therefore, we can conclude that *STC2* mRNA expression is a more promising and reliable blood marker for predicting biological tumor aggressiveness in patients with LSCC. In addition, circulating *STC2* mRNA levels correlated with the expressions of STC2 in tumor tissues in LSCC patients. However, expression levels of the mRNA and protein of *STC1* did not correlate. This might partly explain why *STC2* mRNA expression correlates more closely with biological tumor aggressiveness in LSCC.

In combination with clinicopathological features and other biomarkers of LSCC, STC1 and STC2 expression may be useful for stratifying patients for individual treatments, such as adjuvant chemotherapy or radiotherapy. Some patients demonstrate disease recurrence or metastasis at an early stage after receiving the initial operation. In contrast, others demonstrate long-term survival despite diagnosis at a late stage of disease. This discrepancy may be due to differences in the molecular biology of individual tumors [37]. STC1 or STC2 status may play important roles in tumor biology. Thus, if a patient is found to express high levels of STC1 or STC2, comprehensive treatments, including adjuvant radiochemotherapy, should be recommended. This approach may improve patient survival by eliminating circulating tumor cells and suppressing micrometastases. Moreover, a recent study shows that STC1 and STC2 promote angiogenic sprouting and stimulate vascular endothelial growth factor (VEGF) signaling [38]. Therapies targeted against mediators of angiogenesis, such as VEGF inhibitors, have been shown to prolong the lives of numerous cancer patients. However, primary or secondary resistance to anti-VEGF therapies and multiple side effects remain important challenges to this treatment approach [39]. Hence, alternative, less toxic therapies are needed. STCs represent novel targets against which anti-angiogenesis cancer treatments can be developed.

In the present study, STC1 and STC2 expression did not show similar correlations with the development and progression of LSCC. STC2 expression has more clinical significance for the diagnostic and prognostic assessment of LSCC, whereas STC2 may be more likely to serve as a new molecular therapeutic target. The limitations of this study included the retrospective design and small sample size. In addition, clinical data were not available for existing physical illnesses, occupational information, post-surgical radiotherapy, and other factors that may have influenced the findings of this study. Further investigations in other patient populations or large-scale, well-characterized patient samples may be required to confirm our findings.

In summary, we evaluated the expression status of STCs in tumors and PB of patients with LSCC. Our data indicate that circulating *STC1* and *STC2* mRNA are potentially useful blood markers for LSCC. Most importantly, these results strongly suggest that the STC2 protein may be a valuable biomarker of malignancy in patients with LSCC and an effective prognostic marker of poor post-surgical outcome. The STC1 protein did not show significant correlations with prognosis of patients with LSCC. Further studies are needed to clarify the mechanisms by which STC1 and STC2 are involved in the development and progression of LSCC. These studies will also help clarify the regulatory mechanisms and roles of STCs in tumor angiogenesis, proliferation, and apoptosis.
